# Is Occupation a Good Predictor of Self-Rated Health in China?

**DOI:** 10.1371/journal.pone.0125274

**Published:** 2015-05-07

**Authors:** Zheng Xie, Adrienne N. Poon, Zhijun Wu, Weiyan Jian, Kit Yee Chan

**Affiliations:** 1 School of Public Health, Peking University Health Science Center, Beijing, P. R. China; 2 Rutgers New Jersey Medical School, Newark, United States of America; Kuopio University Hospital, FINLAND

## Abstract

**Background:**

China’s rapidly changing economic landscape has led to widening social inequalities. Occupational status in terms of occupational type and prestige may reflect these socio-structural shifts of social position and be more predictive of self-rated health status than income and education, which may only reflect more gradual acquisitions of social status over time. The goals of this study were to understand the role of occupational status in predicting self-rated health, which is well known to be associated with long-term mortality, as well as compare the occupational status to the other major socioeconomic indicators of income and education.

**Methods:**

Data from the 2010 baseline surveys of the China Family Panel Studies, which utilized multi-stage probability sampling with implicit stratification was used. Logistic regression was used to examine the relationship of various socioeconomic indicators (i.e. occupational status, income, and education) with self-rated health as the primary outcome of interest. A series of models considered the associations of occupational category or occupational prestige with self-rated health.

**Results:**

The final sample consisted of 14,367 employed adults aged 18–60, which was nationally representative of working adults in China. We found that occupation was not a major predictor of self-rated health in China when age, ethnicity, location, marital status, physical and mental health status were controlled for, with the exception of women working in lower grade management and professional jobs (OR = 1.82, 95% CI: 1.03–3.22). In comparison, income followed by education exhibited greater association with self-rated health. The highest income group had the least probability to report poor health (In men: OR = 0.30, 95% CI: 0.21–0.43. In women: OR = 0.44, 95% CI: 0.26–0.73). People educated with junior high school had better self-rated health than those with primary and below education level (In men: OR = 0.62, 95% CI: 0.50–0.75. In women: OR = 0.53, 95% CI: 0.42–0.68). Income, education and occupation were correlated with each other.

**Conclusions:**

Within the context of rapid societal changes in China, income and its implications for greater healthcare access and benefits had the greatest association with self-rated health followed by education. Occupational status was not associated. Occupational categories and prestige should be better adapted to reflect China’s unique sociopolitical and historical context.

## Introduction

China’s rapid development over the past several decades has significantly altered social status including occupational roles. The creation of a workforce based on the needs of the modern society has led to a number of social shifts including a migrating labor force, changing occupational roles for men and women, and new occupational exposures and work conditions that may have negative effects on health status [1–3]. Occupational status, however, may also reflect an individual’s income, education, and social standing, which may contribute to more positive health outcomes as new sources and opportunities have developed [4,5]. Thus the redesign of occupations and their roles in China may be a good indicator of self-rated health by reflecting new differential opportunities, exposures, and access to health resources.

Occupation as an indicator of socioeconomic status (SES) has been studied less in comparison to more commonly used income and education indicators. This is due in part to the complex nature and mechanisms through which occupation may influence health both directly as well as indirectly through other SES indicators. Poor health affects occupational mobility and may lead to selection into less prestigious jobs, while prestigious jobs may be related to more prolonged good health [6]. Occupational prestige, in contrast, reflects a power differential inherent across various occupations [7], which may lead to differential access to resources to improve health. This may be the result of how occupational prestige may impact self-esteem, increase opportunities for social support through expanded networks, and offer subtle benefits [4].

Self-rated health has been found to be correlated with various biomarkers and be an increasingly good predictor of mortality even when other health indicators such as chronic conditions are controlled for [8–12]. This measure has been associated with greater number of deaths due to diabetes, infectious and respiratory diseases, multiple causes, heart disease, stroke, and cancer, with a stronger association in men for a number of diseases [10]. The complex mechanisms by which self-rated health may predict mortality may reflect both physical and mental states related to social and biological mechanisms and may capture subtle bodily status that is difficult to assess through the necessary constraints of empirical studies [12].

Studies on the role of occupational prestige and health status have been conflicting. Fujishiro et al. found higher occupational prestige in the United States to be an independent predictor of better self-rated health [4]. Low occupational prestige has also been associated with metabolic syndrome, greater risk of coronary heart disease in men, and total mortality from any cause, cancer, cardiovascular diseases, and respiratory diseases [13–15]. Other studies have not found consistent results of occupational prestige with self-rated health, nor any association with adherence to dietary guidelines or ambulatory blood pressure [16–20]. As far as occupational classes, those in lower occupational categories may report poorer self-rated health but may have equivalent mortality to those in higher occupational classes [21].

In China, poor self-rated health ranged from 18.5%-70.7%, though these studies were primarily conducted in cities or amongst the elderly [22–33]. Within the local context, self-reported health has been associated with a number of biomarkers (i.e. red blood cells, hemoglobin, aspartate aminotransferase, total cholesterol, triglyceride, low-density lipoprotein, fasting blood glucose) suggesting that it may be a valid indicator of self-rated health [29]. Chronic diseases including diabetes, hypertension, cardiovascular diseases, gastric diseases, respiratory diseases, arthritis, eczema, visual impairment, and mental disorders have all been associated with poor self-rated health [23,29]. Mortality from all causes as well as from stroke and heart disease has also been found to be associated [22,28,34]. Other factors such as life and work pressure, unemployment, lack of insurance, poor sleep quality, living arrangement, loneliness, poor social support mechanisms, poor spirituality, actively seeking health information, and lesser tea consumption have been linked to poorer self-rated health in China [23,26,27,29–31,33,35–39]. Health risks or behaviors including less physical activity, underweight and weight loss, non-alcohol drinking, and early menopause have also been related to poorer self-rated health[29,38].

Given the limited scope of prior studies and large socioeconomic differences across China, comprehensively understanding self-rated health with nationally representative data is needed. Little is known about how occupational status may influence self-rated health in China and thus contribute to long-term outcomes. One study conducted amongst the elderly, however, found an association of occupational history with self-rated health [30]. Given the rapidly changing socioeconomic environment in China, current occupational status and the prestige it affords may better indicate an individual’s health over income and education alone.

Occupation, income, and education, are all proxy measures of SES and may be correlated with each other but are not interchangeable [40]. The pathways through which each SES indicator operate are unique, though may be influenced by other indicators. Occupational status, for example, may reflect the outcome of education and minimize the volatility of income at a point in time [41]. Given that these measures have individual mechanisms despite influence by other SES measures, independently considering each of these indicators will allow greater understanding of how they may operate to determine SES and how it may be related to health status.

Education, for example, is an attained measure and can influence health knowledge, and it may not translate into the financial and occupational resources to allow health access. Income may reflect greater financial access to health resources but does not necessarily imply greater health knowledge and awareness. Self-evaluations inherent in global health status ratings may consider factors negatively impacting health such as occupational exposure and comorbid conditions. Occupational status may reflect a higher societal position attained with any combination of higher income, higher education, and reduced harmful occupational exposures. Thus higher occupational status may be related to higher self-rated health. Occupational status may also reflect recent massive socio-structural shifts of social position and be more predictive of self-rated health than income and education, which may only reflect more gradual acquisitions of social status over time. With the recent explosion of opportunity in China, those earning high income may not be highly educated. Higher education may not necessarily lead to high paying jobs. Occupational status in China is also unique for its context of higher social position for those in state-associated employment. This prestige as well as greater opportunities to access health resources may be reflected in self-rated health.

The aims of this study are to: 1) study the role of occupational prestige and occupation type in China as an independent predictor of health status distinguished from the effects of other SES indicators (i.e. education and income), workplace characteristics (i.e. work hours), and poorer physical and mental health status indicators; and 2) compare occupational status in China to the other major indicators of income and education.

## Methods

### Survey data

**Fig 1:**
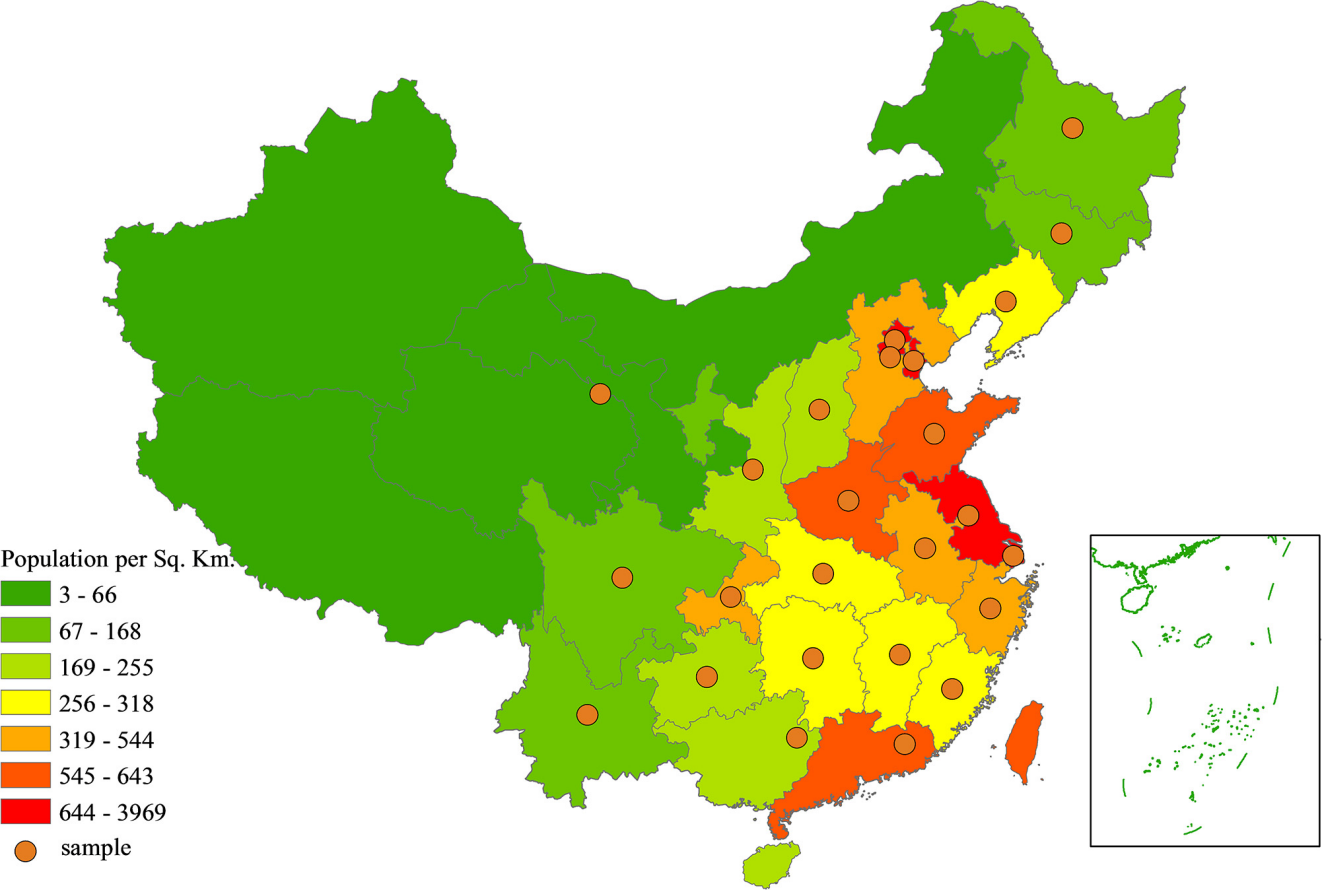
Map of the CFPS Survey Areas.

The data for this study was obtained from the China Family Panel Studies (CFPS), which is a longitudinal prospective cohort study with biannual surveys conducted through Peking University’s Institute of Social Science Survey in collaboration with the Survey Research Center at the University of Michigan [42]. The baseline survey from which this study is drawn was conducted from April 2010 to September 2010, and utilized multi-stage probability sampling with implicit stratification, which requires that the sampling frame be sorted according to specific variables prior to sampling [43]. There was an 84.1% participation rate. The study sample was drawn from 25 provinces representing about 95 percent of the population (Fig 1). Five large provinces or municipalities that included Guangdong, Gansu, Liaoning, Henan, and Shanghai were initially oversampled for comparison according to various regions. The remaining 20 provinces or municipalities were grouped together.

As part of three-stage cluster with probability-proportional to size sampling, county, village, and household subsamples were drawn [44]. In the first stage, 16 counties were sampled from each of the large provinces while 18 counties were sampled from Shanghai. From the other 20 provinces/municipalities, 80 were sampled. This yielded a total of 162 counties or equivalents. Counties or equivalents were considered the primary sampling unit (PSU). In the secondary stage, 649 administrative villages or equivalents were sampled from the selected counties. In the final stage, 19,986 households were sampled from selected villages. Together, this yielded a final nationally representative sample of 42,590 including 33,600 adults and 8990 children after careful weighting [45]. The focus of this study is on the role of occupation with health status in the working age population. Thus inclusion criteria for this study were adults 18–60 years old who earned income from a full-time, temporary, or part-time job from working more than 1 hour in the past week, which yielded a final sample size of 14,367 adults, nationally representative of employed adults in China.

### Ethics Statement

This study has been granted a written ethical exemption by Peking University Biomedical Ethics Committee (IRB00001052-14013-exemption).

### Variables

#### Occupational variables

Occupational categories were initially coded based upon the China General Social Survey (CGSS) and the Internal Migration and Health in China (IMHC) surveys. These categories were then collapsed according to Erikson and Goldthorpe and Portocarero Class Categories (EGP) [46], and then further compressed to the final seven categories for this study: higher grade managers and professionals, lower grade managers and professionals, routine non-manual employees, self-employed, skilled manual workers, semi-skilled and unskilled manual workers, and agricultural workers.

Occupational prestige scores were calculated from Treiman’s Standard International Occupational Prestige Scale (Treiman’s SIOPS), which presumes that across modern societies and social or cultural groups, a level of power and privilege are afforded to individuals according to their occupation [7]. The CFPS questionnaire initially used the Chinese Standard Classification of Occupation (CSCO) which has 8 categories with 595 occupation codes. The CSCO was then converted to the International Standard Classification of Occupation (ISCO-88) which has 10 categories and 390 occupational classifications [47]. Each ISCO-88 occupation has a score according to Treiman’s SIOPS, which was used as the final occupational prestige score in this study. Scales were calculated on a scale of 0–100 for an individual’s primary occupation.

#### Other socioeconomic status variables

Income and education are two major indicators of SES. An individual’s income was collected considering all forms including working wages, off-payroll income, assets, monetary gifts, pension, and any other forms. This continuous variable was divided into quintiles. Highest level of education was asked and grouped as primary and below, junior high school, senior middle school, vocational school, bachelor degree and above.

#### Working conditions variables

Work hours per week, which were grouped as ≤ 44 hours, 45–64 hours, and ≥65 hours, was used to represent a characteristic of working conditions in China that may induce additional stresses and thus influence health status and outcomes [18,29].

#### Health status variables

Several variables were considered to assess a participant’s physical and mental health status. To assess acute illness within the last 2 weeks, the question ‘During the past two weeks, have you felt physically uncomfortable?’ was asked. To assess chronic illness, which has been associated with poor self-rated health in China [22], the question ‘During the past six months, have you had any doctor-diagnosed chronic disease?’ was asked. A larger burden of deaths from chronic disease, including deaths from multiple causes, has previously been found to be associated with poorer health status. Chronic diseases have also been associated with poorer health status in China [10,23,29]. Finally, to assess depressive symptoms, which is associated with poor self-rated health and mortality in China [23,30,37,48], participants were asked if in the past month they felt depressed and cannot cheer up, nervous, agitated or upset and cannot remain calm, hopeless about the future, feel that everything is difficult, or think life is meaningless.

#### Self-rated health variable

Given that self-rated health is increasingly predictive of mortality [9–12], this measure was chosen as the primary outcome variable of interest and was assessed in the survey with the question: ‘How would you rate your health status?’. The responses of healthy, fair, relatively unhealthy, unhealthy, and very unhealthy were dichotomized into a bivariate variable of good (healthy and fair) and poor (relatively unhealthy, unhealthy, very unhealthy) similar to a number of prior studies, which have shown that a dichotomized variable is associated with biomarkers and cardio-metabolic risk factors [8,23].

### Statistical analyses

Data were analyzed using SPSS version 19.0. Descriptive statistics including chi-squares and t-statistics were performed as appropriate. Pearson correlation coefficients were evaluated amongst various SES variables to assess their correlations. A series of logistic regression models were fitted to assess for associations of occupational category or occupational prestige with likelihood of reporting poor self-rated health. Sampling method design was considered in all analyses including accounting for the PSU and applying population weights within the subset of employed adults in China. Given the large significant variation in occupational type between men and women, regression models were fitted separately by gender. All models controlled for age, ethnicity, location, and marital status.

Model 1A assessed if occupational category or occupational prestige had an effect on health status independent of the other SES indicators of income and education. Working conditions local to China were considered in Model 2A, which added work hours. Finally, Model 3 added in physical and mental health variables of having had an acute illness within the past 2 weeks, chronic disease within past 6 months, and depressive symptoms within past month to examine if any differential health status according to occupation were independent of initial poorer health.

In order to compare occupation to income and education as other SES indicators separately in men and women, several models were developed to isolate the effects of income and education on health status. Model 1B assessed if income had an effect on health status independent of education as well as if education had an effect on health status independent of income. Model 2B assessed two separate models for if income or education, respectively, had an association with self-rated health independent of occupation and occupational characteristics. Thus occupational category, occupational prestige, and work hours were added to Model 1B. Finally, Model 3 added in physical and mental health variables to examine if any differential health status according to income or education were independent of initial poorer health.

## Results

Study population characteristics are shown in Table 1. The sample included 5,986 women (41.7%) and 8,381 men (58.3%), with a mean age of 39.0 and a standard error (SE) of 0.2. The large majority (85.9%) were married and of Han Chinese ethnicity (88.8%). Half of women (50.1%) were among those in the lowest income levels. About 70.8% have a junior high school or lower level of education. The mean occupational prestige score was 40.3 (SE 0.2). In terms of SES indicators, income exhibited a strong correlation with occupation (r = 0.616, p<0.001) as well as education (r = 0.489, p<0.001). Occupation was also correlated with education (r = 0.575, p<0.001).

**Table 1:** Descriptive characteristics stratified by gender of working age adults age 18–60 in China.

Characteristic	Male	Female	Total
	N (%) [SE]^∆^ (n = 7846)	N (%)[SE](n = 6521)	N (%)[SE](n = 14367)
Age, mean [SE]	39.2 [0.2]	38.8 [0.3]	39.0 [0.2]
			
Married	6,831 (84.4%) [0.6%]	5,870 (88.0%) [0.7%]	12,701 (85.9%) [0.5%]
			
Han Chinese	7,166 (89.7%) [1.9%]	5,876 (87.7%) [2.2%]	13,042 (88.8%) [2.0%]
			
*Income*			
Lowest 20%	872 (9.5%) [0.7%]	1,961 (25.9%) [1.4%]	2,833 (16.3%) [1.0%]
Lower 20%	1,316 (16.3%) [1.0%]	1,539 (24.2%) [1.2%]	2,855 (19.6%) [1.0%]
Medium 20%	1,660 (21.0%) [0.7%]	1,187 (20.8%) [0.8%]	2,847 (20.9%) [0.6%]
Higher 20%	1,898 (26.4%) [0.8%]	955 (16.3%) [0.8%]	2,853 (22.2%) [0.7%]
Highest 20%	2,057 (26.8%) [1.4%]	796 (12.8%) [1.1%]	2,853 (21.0%) [1.3%]
			
*Education*			
Primary and below	2,783 (33.0%) [1.5%]	3,292 (45.8%) [1.9%]	6,075 (38.3%) [1.6%]
Junior high school	2,799 (36.4%) [0.9%]	1,688 (27.1%) [1.0%]	4,487 (32.5%) [0.9%]
Senior middle school	1,322 (17.2%) [0.7%]	786 (13.6%) [0.8%]	2,108 (15.7%) [0.7%]
Vocational School	555 (8.0%) [0.6%]	440 (7.7%) [0.6%]	995 (7.9%) [0.5%]
Bachelor degree or above	384 (5.4%) [0.5%]	311 (5.7%) [0.7%]	695 (5.6%) [0.5%]
			
*Geographic Region*			
Municipality (Beijing, Chongqing, Shanghai, Tianjin)	929 (6.0%) [1.5%]	707 (5.5%) [1.4%]	1,636 (5.8%) [1.4%]
East	2,262 (27.8%) [2.8%]	1,841 (27.2%) [2.8%]	4,103 (27.6%) [2.8%]
Central	2,563 (40.4%) [3.4%]	2,026 (38.6%) [3.4%]	4,589 (39.6%) [3.4%]
West	2,092 (25.8%) [3.3%]	1,947 (28.8%) [3.5%]	4,039 (27.0%) [3.4%]
			
Self-rated poor/fair health	672 (8.0%) [0.4%]	732 (12.2%) [0.7%]	1,404 (9.8%) [0.5%]
			
Occupational prestige, mean [SE]	40.3 [0.2]	40.3 [0.2]	40.3 [0.2]
			
*Occupational categories*			
High grade managers and professionals	619 (7.9%) [0.5%]	303 (5.1%) [0.4%]	922 (6.7%) [0.4%]
Low grade managers and professionals	511 (7.0%) [0.5%]	527 (9.8%) [0.7%]	1,038 (8.2%) [0.5%]
Routine non-manual employees	420 (5.6%) [0.4%]	695 (12.5%) [0.9%]	1,115 (8.5%) [0.5%]
Self-employed	929 (14.0%) [0.7%]	416 (7.3%) [0.4%]	1,345 (11.2%) [0.5%]
Skilled manual workers	962 (14.0%) [0.9%]	386 (6.2%) [0.5%]	1,348 (10.8%) [0.6%]
Semi-skilled and unskilled workers	1,035 (12.5%) [0.8%]	539 (8.6%) [0.7%]	1,574 (10.9%) [0.7%]
Agricultural workers	3,086 (35.3%) [2.2%]	3,488 (48.0%) [2.4%]	6,574 (40.6%) [2.3%]
			
*Hours worked per week*			
≤ 44 hours	2,753 (35.0%) [1.1%]	2,926 (43.6%) [1.3%]	5,679 (38.6%) [1.1%]
45–64 hours	3,011 (40.0%) [0.8%]	2,148 (36.3%) [1.0%]	5,159 (38.4%) [0.8%]
≥ 65 hours	1,873 (25.1%) [0.9%]	1,205 (20.1%) [0.9%]	3,078 (23.0%) [0.8%]
			
Acute illness in last 2 weeks	1,596 (18.9%) [0.7%]	1,859 (25.2%) [0.9%]	3,455 (21.5%) [0.7%]
			
Diagnosed chronic disease	828 (9.2%) [0.4%]	848 (10.9%) [0.5%]	1,676 (9.9%) [0.4%]
			
Depressive symptoms in last month	974 (11.8%) [0.6%]	1065 (14.7%) [0.8%]	2,039 (13.0%) [0.6%]
			

^**∆**^ SE: standard error

Weighted proportions accounting for sampling design were used to calculate proportions and standard errors.

In terms of occupational type, agriculture consisted of the largest component of the labor force (40.6%) followed by self-employed workers (11.2%) (Table 1). Higher grade managers and professionals had the highest levels of occupational prestige in China (mean 63.3, SE 0.4) (Table 2), followed by lower grade managers and professionals (mean 55.0, SE 0.3) and agricultural workers (mean 39.7, SE 0.1). Semi-skilled and unskilled manual workers had the lowest levels of prestige (mean 29.9, SE 0.2), followed by self-employed workers (mean 34.1, SE 0.3).

**Table 2:** Occupational prestige of various occupational categories and distribution of work hours.

Occupational category	Work Hours			
	Mean (SE^∆^)	≤ 44 hours (N, %) ***	45–64 hours (N, %) ***	≥ 65 hours (N, %) ***
High grade managers and professionals	63.5 (0.4)	393 (40.8%) [1.7%]	328 (38.9%) [1.8%]	172 (20.3%) [1.5%]
Low grade managers and professionals	55.0 (0.3)	541 (54.6%) [2.2%]	348 (34.0%) [1.8%]	108 (11.4%) [1.1%]
Routine non-manual employees	36.4 (0.2)	420(35.4%) [2.0%]	465 (47.9%) [2.0%]	171 (16.7%) [1.1%]
Self-employed	34.1 (0.3)	254(19.4%) [1.1%]	450 (36.4%) [1.5%]	575 (44.1%) [1.5%]
Skilled manual workers	37.0 (0.2)	291(19.1%) [1.6%]	629(51.1%) [1.7%]	388 (29.8%) [1.6%]
Semi-skilled and unskilled workers	29.9 (0.2)	363 (21.5%) [1.3%]	664 (46.4%) [1.3%]	474 (32.2%) [1.5%]
Agricultural workers	39.7 (0.1)	3,331(51.5%) [2.0%]	2,137 (32.8%) [1.2%]	999(15.7%) [1.5%]

^**∆**^ SE: standard error

***p<0.001. Weighted proportions accounting for sampling design were used to calculate proportions and standard errors.

Roughly equal proportions of the labor force work ≤44 hours a week (38.6%) and 45–64 hours a week (38.4%) (Table 1). In considering number of work hours according to various occupational categories and prestige scores (Table 2), those working in occupations with low prestige scores reported longer work hours while those with higher prestige scores reported fewer work hours. Self-employed workers comprised the majority of those working ≥65 hours, while non-manual, skilled manual, and semi-skilled and unskilled workers comprised a majority of those working 45–64 hours. In contrast, a majority of high and low grade managers and professionals as well as agricultural workers reported working fewer than 44 hours. Poor health was reported by 9.8% of men with more women reporting poor health status (12.2%). As far as physical and mental correlates, about 21.5% reported acute illness within the last 2 weeks (18.9% of men and 25.2% of women). About 9.9% reported having had a diagnosed chronic disease within the past 6 months (9.2% of men and 10.9% of women). Additionally, 13.0% had a depressive symptom within the past month (11.8% of men and 14.7% of women).

In considering the association of poor self-rated health with SES indicators, working conditions, and physical and mental health correlates, occupational prestige was not associated with reporting poor self-rated health (Table 3). Occupational categories were associated with poor self-rated health, with a relative gradient towards those in lower grade occupations reporting poorer self-rated health. The only exceptions were lower grade managers and professionals in both genders and self-employed females who all experienced poorer self-rated health. Of note (Tables 2 and 3), both male and female agricultural workers reported the poorest self-rated health with 12.1% and 19.2%, respectively, even though agricultural work is associated with prestige levels (mean 39.7, SE 0.1), higher than most occupations in China excluding managerial and professional levels. Those with the lower income gradients reported poorer self-rated health along clear gradients, with the exception of high school educated females (Table 3). Hours worked per week was associated with self-rated health in women, but was not significant in men. Acute illness in the past 2 weeks, chronic disease, and depressive symptoms were all associated with poorer self-rated health.

**Table 3:** Descriptive statistics of poor health status by various SES indicators, stratified by gender.

SES indicator	Male	Female
***Occupational prestige*, *mean***	**n**	**n**
***Poor health status***	39.7	39.5
***Good health status***	40.3	40
	t = 1.3, p = 0.180	t = 1.6, p = 0.107
***Occupation categories***	**N (%) [SE** ^**∆**^ **]**	**N (%) [SE]**
High grade managers and professionals	29 (4.4%) [0.7%]	13 (4.0%) [1.2%]
Low grade managers and professionals	36 (5.1%) [0.8%]	34 (6.8%) [1.0%]
Routine non-manual employees	23 (5.9%) [1.2%]	30 (4.0%) [0.6%]
Self-employed	56 (5.8%) [0.7%]	35 (7.0%) [1.1%]
Skilled manual workers	64 (5.5%) [0.6%]	24 (4.7%) [1.0%]
Semi-skilled and unskilled workers	76 (7.0%) [0.7%]	53 (7.4%) [0.9%]
Agricultural workers	415 (12.1%) [0.8%]	781 (19.2%) [1.2%]
	χ^2^ = 107.3, p<0.001	χ^2^ = 255.6, p<0.001
***Income***		
Lowest 20%	150 (15.8%) [1.8%]	469 (20.0%) [1.5%]
Lower 20%	195 (13.5%) [1.0%]	294 (17.0) [1.1%]
Medium 20%	151 (8.1%) [0.6%]	99 (6.1%) [0.6%]
Higher 20%	122 (5.8%) [0.5%]	63 (5.6%) [0.7%]
Highest 20%	95 (4.0%) [0.4%]	40 (5.5%) [0.7%]
	χ^2^ = 184.4, p<0.001	χ^2^ = 231.2, p<0.001
***Education***		
Primary and below	388(13.0%) [0.9%]	739 (19.2%) [1.2%]
Junior high school	197 (5.9%) [0.4%]	143 (6.7%) [0.6%]
Senior middle school	92 (5.8%) [0.6%]	67 (7.4%) [0.9%]
Vocational School	30 (5.21%) [0.9%]	16 (3.8%) [0.9%]
Bachelor degree or above	12 (3.6%) [0.8%]	18 (5.9%) [1.3%]
	χ^2^ = 141.4, p<0.001	χ^2^ = 230.3, p<0.001
***Hours worked per week***		
≤ 44 hours	244 (7.9%)[0.6%]	468(13.3%)[1.0%]
45–64 hours	277(7.8%)[0.5%]	296(10.7%)[0.8%]
≥ 65 hours	169(8.3%)[0.7%]	179(13.0%)[1.2%]
	χ^2^ = 0.4, p = 0.826	χ^2^ = 7.4, p = 0.025
**Acute illness in last 2 weeks**	442(25.3%)[1.1%]	654(30.2%)[1.5%]
	χ^2^ = 789.3, p<0.001	χ^2^ = 610.6, p<0.001
**Diagnosed chronic disease**	254(31.5%)[1.5%]	358(35.4%)[2.2%]
	χ^2^ = 631.8, p<0.001	χ^2^ = 369.4, p<0.001
**Depressive symptoms in last month**	251(22.8%)[1.8%]	392(31.1%)[1.9%]
	χ^2^ = 332.5, p<0.001	χ^2^ = 345.6, p<0.001

^**∆**^ SE: standard error.

Weighted proportions accounting for sampling design were used to calculate proportions and standard errors.

In the multivariate models considering occupational status as an SES indicator, higher occupational prestige was not associated with poorer self-rated health in any of the models for both men and women (Tables 4 and 5). None of the occupational categories were associated with poorer self-rated health in males. In Model 2A for females when income, education, and work hours were controlled for, agricultural workers were more likely to have poor self-rated health (OR = 1.70, 95% CI: 1.01–2.87). Lower grade managerial and professional women exhibited poorer self-rated health (OR = 1.73, 95% CI: 1.03–2.89) in Model 2A, which persisted when physical and mental correlates were controlled for (OR = 2.03, 95% CI: 1.02–4.06).

**Table 4:** Association of SES indicators with poor health status in men.

** **	**Model 1A**		**Model 2A**		**Model 3**	
	OR ∆	95% CI ∆ ∆	OR	95% CI	OR	95% CI
***Occupational prestige***	1.00	(0.98–1.01)	1.00	(0.98–1.01)	1.00	(0.99–1.01)
***Occupation categories (ref*. *High grade managers and professionals)***						
Low grade managers and professionals	1.11	(0.73,1.69)	1.13	(0.74,1.73)	0.93	(0.59,1.48)
Routine non-manual employees	1.28	(0.74,2.19)	1.29	(0.75,2.23)	1.26	(0.68,2.31)
Self-employed	1.01	(0.69,1.49)	1.00	(0.68,1.48)	0.88	(0.57,1.35)
Skilled manual workers	1.13	(0.82,1.57)	1.13	(0.81,1.57)	1.08	(0.72,1.59)
Semi-skilled and unskilled workers	1.27	(0.90,1.80)	1.27	(0.90,1.79)	0.99	(0.67,1.47)
Agricultural workers	0.97	(0.70,1.36)	1.00	(0.71,1.39)	1.08	(0.74,1.57)
	**Model 1B**		**Model 2B**		**Model 3**	
	OR	95% CI	OR	95% CI	OR	95% CI
***Income (ref*. *Lowest 20%)***						
Lower 20%	0.78	(0.60,1.02)	0.76*	(0.58,0.99)	0.68**	(0.51,0.89)
Medium 20%	0.50***	(0.39,0.65)	0.46***	(0.35,0.61)	0.45***	(0.34,0.61)
Higher 20%	0.42***	(0.31,0.55)	0.40***	(0.29,0.54)	0.44***	(0.31,0.60)
Highest 20%	0.29***	(0.21,0.39)	0.28***	(0.19,0.37)	0.30***	(0.21,0.43)
						
***Education (ref*. *Primary and below)***						
Junior high school	0.60***	(0.49,0.74)	0.28***	(0.48,0.71)	0.62***	(0.50,0.75)
Senior middle school	0.66**	(0.51,0.85)	0.64**	(0.50,0.83)	0.65**	(0.48,0.86)
Vocational School	0.88	(0.57,1.36)	0.91	(0.54,1.54)	0.79	(0.42,1.49
Bachelor degree or above	0.67	(0.41,1.10)	0.71	(0.40,1.29)	0.55	(0.27,1.10)

∆ OR: odds ratio, ^**∆ ∆**^ CI: confidence interval.

*p<0.05

**p<0.01

***p<0.001

All models adjusted for age, ethnicity, location, and marital status. Model 1A included the variables of occupational prestige, occupation, income, and education, which examined the association between occupational prestige/occupation and poor health status while controlling for income and education. Model 1B included income and education aiming to examine the association between income with poor health status while controlling for education and vice versa. Model 2A added work hours to Model 1A. Model 2B added occupation type, prestige, and work hours to Model 1B. Finally, Model 3 added physical and mental health variables to Model 2B.

**Table 5:** Associations of SES indicators with poor health status in women.

	**Model 1A**		**Model 2A**		**Model 3**	
	OR ^**∆**^	95% CI ^**∆ ∆**^	OR	95% CI	OR	95% CI
***Occupational prestige***	1.00	(0.99–1.01)	1.00	(0.99–1.02)	1.01	(1.00–1.02)
						
***Occupation categories (ref*. *High grade managers and professionals)***						
Low grade managers and professionals	1.64	(0.98,2.74)	1.73*	(1.03,2.89)	1.82*	(1.03,3.22)
Routine non-manual employees	1.01	(0.57,1.81)	1.05	(0.59,1.87)	0.83	(0.45,1.54)
Self-employed	1.06	(0.58,1.93)	1.00	(0.54,1.82)	0.87	(0.44,1.73)
Skilled manual workers	0.85	(0.43,1.66)	0.85	(0.43,1.66)	0.82	(0.37,1.82)
Semi-skilled and unskilled workers	1.29	(0.75,2.22)	1.31	(0.76,2.24)	1.20	(0.63,2.27)
Agricultural workers	1.61	(0.95,2.72)	1.70	(1.01,2.87)	1.63	(0.89,3.02)
	**Model 1B**		**Model 2B**		**Model 3**	
	OR	95% CI	OR	95% CI	OR	95% CI
***Income (ref*. *Lowest 20%)***						
Lower 20%	0.88	(0.72,1.06)	0.89	(0.74,1.08)	0.93	(0.77,1.11)
Medium 20%	0.36***	(0.29,0.45)	0.42***	(0.33,0.53)	0.45***	(0.35,0.58)
Higher 20%	0.41***	(0.30,0.57)	0.52***	(0.37,0.74)	0.55**	(0.37,0.81)
Highest 20%	0.40***	(0.26,0.61)	0.51**	(0.31,0.82)	0.44***	(0.26,0.74)
						
***Education (ref*. *Primary and below)***						
Junior high school	0.52***	(0.42,0.64)	0.52***	(0.42,0.65)	0.53***	(0.42,0.68)
Senior middle school	0.76	(0.58,1.01)	0.88	(0.65,1.19)	0.93	(0.66,1.31)
Vocational School	0.59	(0.32,1.07)	0.69	(0.36,1.29)	0.76	(0.42,1.40)
Bachelor degree or above	0.98	(0.58,1.68)	1.08	(0.59,1.96)	0.91	(0.46,1.83)

^**∆**^ OR: odds ratio, ^**∆ ∆**^ CI: confidence interval.

*p<0.05

**p<0.01

***p<0.001

All models adjusted for age, ethnicity, location, and marital status. Model 1A included the variables of occupational prestige, occupation, income, and education, which examined the association between occupational prestige/occupation and poor health status while controlling for income and education. Model 1B included income and education aiming to examine the association between income with poor health status while controlling for education and vice versa. Model 2A added work hours to Model 1A. Model 2B added occupation type, prestige, and work hours to Model 1B. Finally, Model 3 added physical and mental health variables to Model 2B.

In comparison, there were strong associations of income with poorer health status (Table 4). In both genders, those with higher income were less likely to experience poorer self-rated health when education, occupation, and physical and mental health correlates were all controlled for. Education experienced less clear gradients. In men, those with education through senior high school were less likely to experience poorer self-rated health than those with only primary education. This association persisted through all models when income, occupation, and physical and mental health correlates were all controlled for. Women educated through middle school were less likely to have poorer self-rated health in all models.

## Discussion

In China, occupation did not appear to have a strong association with self-rated health, especially in comparison to income and education. Similar to previous findings in other countries [16–18], there was no association of occupational prestige with self-rated health in either gender. This may be due to the large variations within occupational categories, where some occupations earn disproportionately higher income, which is more likely to be associated but difficult to distinguish within the occupational categorizations. Occupational classifications and prestige may also be further defined according to systems unique to China, such as through work units and their associated political prestige in the Mao era, which may be less suitable to categorization by international standards. Those working in state-owned enterprises, for example, have higher levels of prestige than privately owned enterprises given the politically associated social mobility [3,49,50]. Liu and Zhang, however, found a much clearer graded relationship between occupational type and self-rated health [30]. This may be because occupational history before age 60 was assessed amongst the eldest of the elderly, and thus evaluated a time when occupational status was much less subject to fluctuations in comparison to the current socioeconomic climate with rapidly changing job opportunities.

Given China’s explosion of economic opportunities and associated rapid development, the relationship of occupation with income and education may not be as well defined. With the continuing high level of social mobility afforded by a rapidly evolving economy, many who earn high income may not be in high grade occupations or have comparable education levels expected with high income. The correlations of income with both education and occupation as well as the association of income with self-rated health suggest the more subtle influences of education and occupation on higher social status and self-rated health. The complexities are intricate and should be further explored, with consideration of how best to capture the rapidly shifting occupational environment and intricacies of work strata in a meaningfully relevant SES indicator within the Chinese context. Such an indicator should consider factors such as power, *hukou* (residency), public/private enterprise sectors, and stigmatization of certain fields of work [3,51,52]. These factors are unique to China’s political and economic environment. Historically, a person’s *hukou* was tied to their residency and occupation under the communist-controlled economy. This economy has and continues to include public government owned enterprises that maintain a significant sociopolitical position within China. Finally given this historical context as well as the current market-driven economy atmosphere, fields of work that may be stigmatized or be considered highly may need to be further elucidated. Higher levels of prestige for agricultural work, for example, may be more unique to China.

Although occupational prestige was not associated with health status, occupational categories exhibited a greater association for women. Female agricultural workers were more likely to have poorer health status independent of income, education and work hours. As China rapidly develops, increasingly more men are working in cities or establishing family businesses leaving more women to independently manage farms [53–55]. Traditionally, women have experienced a greater burden of family responsibilities as well, which is becoming more critical with a large aging population but smaller young population due to the one-child policy [56,57]. These burdens are now being expanded with greater share of physically strenuous agricultural work while many men seek work in cities and factories, and may possibly be related to the high prevalence of mood disorders and suicide amongst young women in rural areas [58–60]. Similarly in this study, we noted that more women, including women with poorer self-rated health, reported symptoms of depression in the past month than men. Given the association of poorer self-rated health with mortality [9,10,12], this additional burden may contribute to poorer long-term outcomes. In particular, the high level of physical demands associated with agricultural work may negatively influence the perception of self-rated health of many of these women.

Women who are lower grade managers and professionals were also more likely to report poorer self-rated health after controlling other factors. This may be due to external factors such as work and social pressures and limited opportunity for women to seek promotion to the highest levels [61,62]. Our data showed that a greater proportion of men were higher grade managers or professionals. Men in lower grade managerial or professional positions may have greater opportunity for promotion and may consider themselves healthier and physically fit to meet the demands of higher grade jobs and thus report better self-rated health. Fewer women were in these high grade professional positions, while larger proportions were low grade managers and professionals. Thus the self-rated health of these women in lower grade professions may reflect a perceived lack of mobility and further opportunity while working in positions with high pressure and stress. Further research is needed to understand the mechanisms by which men and women perceive their health status as a function of perceived occupational mobility.

The linkages among gender, poor health status, and mortality in China are unclear, though women may rate their health status as worse even though these differences are not significant [24]. Evidence from other countries suggests that women who report poorer self-rated health may experience lower mortality [63]. Elderly women in China may have a higher burden of chronic disease [24], which may influence their perceptions and self-assessments of their health. Future studies should consider the multiplicative framework under which women in China assess and evaluate their health [29].

Income in comparison to occupation was more associated with self-rated health along a relative gradient independent of each other, occupational factors, and physical and mental health correlates with the exception of lower income women. This is consistent with previous studies that found an association with income level and self-rated health amongst Chinese populations [23,39,64,65]. Income may both directly and indirectly influence how individuals in China may evaluate and report their health status. China over the past several decades had increasingly shifted towards a money-driven economy where high income affords a high amount of power, privilege, and accessibility within modern Chinese society, including for better health care [66–71]. Higher levels of income may also influence self-esteem and thus positively rate higher an individual’s self-perception of health status. Additionally, higher income may allow for better access to high quality health care, more opportunities to learn health information, and allow for better living conditions, all of which may directly and indirectly improve health status [39,66,72,73].

Our finding that those with lower education levels were more likely to independently exhibit poorer self-rated health was consistent with prior studies in China. This may be because those with lower education levels may have worse physical health or functional status, while those in the highest levels have greater health expectations [24,30,33,38,39]. Of note, income and education were strongly correlated. Education, however, did not show as strong a gradient as income and may partially contribute through earning higher income instead. Education and occupation were correlated as well, suggesting that those with higher education may have a higher grade occupation or those with higher occupation are better educated. It was notable, however, that a majority of employed women are concentrated in the lowest-paid agricultural work and that a majority of women have the lowest levels of education in China. Strengthening programs to increase the education and employment opportunities for women may serve to indirectly improve their health status.

In addition, occupations that require more education, have higher social esteem, and have better income may be safer [18], which may be avenues by which education and income indirectly contribute to higher grade occupation and better health status. Self-rated health may reflect income and education through in ways that are more directly perceived [63]. Occupation slightly mitigated the effects of both income and education, which supports the complex interaction amongst various SES factors.

Other studies in China had found much higher levels of poor self-rated health [22–33], while we found a much lower level of poor self-rated health (9.8%). A majority of the prior studies were conducted in cities or included the middle-aged and elderly population who may report poorer health status as age increases and health expectations decrease [29,32,33,38,39]. The distribution of poorer health status by age in our study shows a similar trend towards poorer self-rated health with increasing age (unpublished data). Furthermore, we only considered the employed population, who are more likely to have better health status [32,39].

Although self-rated health may vary across different sociocultural groups, how health is understood may reflect how individuals within these groups may evaluate the severity, consequences, and prognosis of diseases and conditions [12]. To our knowledge, this is the first study nationally representative of employed adults in China to consider the influence of complex interrelated social class positioning with self-rated health, which may indicate the likelihood of future mortality amongst employed adults with poor ratings of their health.

Future studies should involve further evaluation of the association of self-rated health in China with objective biomarkers and long-term mortality outcomes to better understand the utility of self-rated health interpretation. Although a number studies in other countries have found such associations [8–11], contextual factors may not allow self-rated health to be directly comparable across different populations [12]. Only one study from China evaluated the relationship of biomarkers to self-rated health but was conducted in the urban context [29]. Additionally, given the rapid development in China over time, future studies should also consider how changing socioeconomic status may affect self-reported health and mortality. Furthermore, the subtle mechanisms through which occupation may influence income and education should be further explored. In studies of China, occupation may not be the best SES indicator given a rapidly developing economy. Should the economy reach a more relatively steady state, occupational status may be more appropriately applied in terms of understanding self-rated health. In a rapidly developing economy, there may be more diverse opportunities for people with varying educational backgrounds to obtain greater wealth and living conditions given a more fluid social status. Higher education perhaps has not yet transformed into a marker of greater opportunity and resources in a shifting economy. It may rather be a stable indicator of self-rated health in more socially static developed economies where higher education generally is associated with higher social status. Income as an indicator within a rapidly developing economic context, however, may be most stably used since it may indicate the greatest prosperity within an expanding economy and thus lead to greater access of health resources.

Several limitations of this study should be considered. First, although this study is generalizable to a majority of the population in China, it may not be directly comparable to populations in other countries, especially given the unique sociocultural contexts through with the assessment of self-rated health operates [12]. Second, this study focuses only on the working adult population, excluding those who are not working or unemployed, who are more likely to have poorer health status [32,39], and the young and elderly who may exhibit differential health status with differential influence from socioeconomic status indicators. The elderly in particular report poorer self-rated health [29,32,33,38,39]. Third, income rather than household income was used, which may underestimate an individual’s socioeconomic status. Fourth, physical and mental health correlated, which were subjectively gathered are subject to the same limitations of evaluation using a self-reported framework [12], especially without clear distinctions between clinically verified and subjective reports. This likely contributed to the variable mitigation of the models once other health factors were accounted for. Fifth, self-rated health status may not equate to the same level of health indicators and outcomes for differing education levels [74,75]. Additionally, the dichotomization of self-rated health makes it difficult to capture the complexities of self-rated health across gradients. Finally, the subtle and complex interactions amongst various markers of socioeconomic status are by nature difficult to assess through variables, though we attempted to assess some of the less complex interactions in this study. Despite these limitations, this study is the first nationally representative study of employed adults in China to assess self-rated health status and its association with socioeconomic positioning. It builds upon the current understanding of the applicability of various socioeconomic status indicators in China and provides a more in depth understanding of self-rated health in a younger working age population.

## Conclusion

In conclusion, occupational status including classifications and prestige were not the best predictors of poorer health status in China in comparison to income and education. These occupational indicators may not be suitable to China’s context given the sociopolitical history around labor and the massive ongoing shifts around occupation and opportunity. Existing categorizations may not be sensitive enough to capture these fluctuations and their influences on health status. Occupation, however, does correlate with both income and education demonstrating the complex interactions amongst these SES indicators. The mechanisms however are less direct. Low income demonstrated the strongest association with poorer self-rated health with a clear social gradient in both genders that could not be explained by other SES indicators or poorer health indicators. Those with lower income are distributed across varying occupational groups. Social and health policy targeting those with lower income should also attempt to reach those disadvantaged across occupational groups. We also found that women have lower income level than men. These gender disparities should be considered when designing health policy and programs to target women, especially those in rural areas or low grade managers and professionals who may be at greater risk for poor health. Our research shows that when understanding China’s society, occupation is not a good indicator for social stratification, different from other societies, and possibly due to the rapid pace of development in China’s economy. These factors should be given consideration when designing global health program or interventions. Greater effort should be targeted towards improving the health of those with lower income and education levels to improve health status and outcomes.
